# Characterization of Botulinum Neurotoxin Type A Neutralizing Monoclonal Antibodies and Influence of Their Half-Lives on Therapeutic Activity

**DOI:** 10.1371/journal.pone.0012416

**Published:** 2010-08-26

**Authors:** Christelle Mazuet, Julie Dano, Michel R. Popoff, Christophe Créminon, Hervé Volland

**Affiliations:** 1 Unité des Bactéries anaérobies et Toxines, Institut Pasteur, Paris, France; 2 CEA, iBiTecS, Service de Pharmacologie et d'Immunoanalyse, Gif sur Yvette, France; Max Planck Institute for Infection Biology, Germany

## Abstract

Botulinum toxins, i.e. BoNT/A to/G, include the most toxic substances known. Since botulism is a potentially fatal neuroparalytic disease with possible use as a biowarfare weapon (Centers for Disease Control and Prevention category A bioterrorism agent), intensive efforts are being made to develop vaccines or neutralizing antibodies. The use of active fragments from non-human immunoglobulins (F(ab')_2_, Fab', scFv), chemically modified or not, may avoid side effects, but also largely modify the *in vivo* half-life and effectiveness of these reagents. We evaluated the neutralizing activity of several monoclonal anti-BoNT/A antibodies (mAbs). F(ab')_2_ fragments, native or treated with polyethyleneglycol (PEG), were prepared from selected mAbs to determine their half-life and neutralizing activity as compared with the initial mAbs. We compared the protective efficiency of the different biochemical forms of anti-toxin mAbs providing the same neutralizing activity. Among fourteen tested mAbs, twelve exhibited neutralizing activity. Fragments from two of the best mAbs (TA12 and TA17), recognizing different epitopes, were produced. These two mAbs neutralized the A1 subtype of the toxin more efficiently than the A2 or A3 subtypes. Since mAb TA12 and its fragments both exhibited the greatest neutralizing activity, they were further evaluated in the therapeutic experiments. These showed that, in a mouse model, a 2- to 4-h interval between toxin and antitoxin injection allows the treatment to remain effective, but also suggested an absence of correlation between the half-life of the antitoxins and the length of time before treatment after botulinum toxin A contamination. These experiments demonstrate that PEG treatment has a strong impact on the half-life of the fragments, without affecting the effectiveness of neutralization, which was maintained after preparation of the fragments. These reagents may be useful for rapid treatment after botulinum toxin A contamination.

## Introduction

Seven serologically distinct botulinum toxins, BoNT/A to/G, are produced by different strains of the Gram-positive, spore-forming anaerobic bacterium *Clostridium botulinum*. These neurotoxins are the most toxic substances known, being, for example, 100,000 times more toxic than sarin [1]. Three main forms of human botulism generally associated with toxinotypes A, B and E have been described: i) foodborne botulism, ii) intestinal colonization (infant botulism and foodborne illness in adults) due to toxin production in the intestine after ingestion of *Clostridium botulinum* spores, and iii) wound botulism [2]. Botulism cases are rare but can be life-threatening and the recovery period requires intensive care and can take several months. Vaccination against botulism is available and consists of a long-lasting inoculation protocol which has only been used for people at high risk of exposure and not for whole populations. Since no drugs allow prevention or treatment, toxin-neutralizing antibodies were developed for prophylactic or therapeutic treatment [3]. These antitoxins were obtained after immunization of several species: horse [4], goat [5], mouse [6]–[7], and human [8]. However, the use of antitoxin from nonhomologous species can generate side effects including anaphylactic shock [9] and the production of human anti-species antibodies. Moreover, human antitoxin antibodies from immunized volunteers also have limitations related to their small-scale production and the risk of infectious disease transmission. Different strategies have been developed to circumvent these limitations using phage display libraries from immunized mice or humans [10], [11] or using immunoglobulin fragments, like F(ab')_2_ [12], [13], which are less immunogenic. However, these fragments have short *in vivo* half-lives, which must be taken into account considering the expected duration of antitoxin activity. Several reports have shown that linking polyethylene glycol (PEG) molecules to F(ab')_2_ fragments (pegylated fragments) can overcome this problem by extending *in vivo* half-life [14]–[17].

In the present study, the neutralizing potency of 14 monoclonal antibodies (mAbs) raised against BoNT type A was estimated. F(ab')_2_ fragments from the most efficient mAbs were then produced and further modified by PEG treatment. The neutralizing effects and the *in vivo* half-lives of the fragments, pegylated or not, were characterized before finally evaluating their efficiency for therapeutic treatment of mice challenged with BoNT/A.

## Materials and Methods

### Reagents

Unless otherwise stated, all reagents were from Sigma (St. Louis, MO). *C. botulinum* cultures require handling precautions, due to their toxicity. Appropriate laboratory attire should be worn, including a lab coat, gloves and safety glasses. BoNT-contaminated materials were inactivated by immersion in 5% sodium hypochlorite solution for 24 h. *C. botulinum* strains were grown in TGY (30 g/l trypticase; 5 g/l glucose; 20 g/l yeast extract, 0.5 g/l cysteine hydrochloride; pH 7.5) in anaerobic conditions for 4 days at 37°C. The cultures were acidified at pH 3.5 with sulfuric acid, centrifuged, and the pellet was extracted with 0.2 M sodium phosphate buffer pH 6.0 as previously described [18]. The extracted material constitutes the toxin stock.

Recombinant Hc BoNT/A1 fragment, corresponding to the binding domain of the neurotoxin type A1, was prepared as previously described [19].

N-Hydroxysuccinimidyl ester of methoxy poly(ethylene glycol) butanoic acid (abbreviated as NHS-PEG) corresponding to a 30 kDa linear PEG was from Nektar Therapeutics (Huntsville, AL).

For immunoassays, all reagents were diluted in EIA buffer (0.1 M phosphate buffer pH 7.4 containing 0.15 M NaCl, 0.1% bovine serum albumin (BSA) and 0.01% sodium azide). Plates were washed with washing buffer (0.01 M phosphate pH 7.4 buffer containing 0.05% Tween 20). Ellman's medium used to reveal the activity of acetylcholinesterase (AChE), an enzyme used as marker in the immunoassays, comprised a mixture of 7.5 10^−4^ M acetylthiocholine iodide (enzyme substrate) and 2.5 10^−4^ M 5,5′-dithiobis(2-nitrobenzoic acid) (DTNB) (reagent for thiol colorimetric measurement) in 0.1 M phosphate buffer, pH 7.4. Enzymatic activity was expressed in Ellman units (EU). One EU is defined as the amount of enzyme producing an absorbance increase of one unit during 1 min in 1 ml of medium, for an optical path length of 1 cm; it corresponds to about 8 ng of enzyme.

### Apparatus

Immunometric assay was performed using Titertek microtitration equipment from Labsystem (Helsinki, Finland), including an automatic plate washer (Washer 120) and an automatic plate reader (Multiskan BICHROMATIC). 96-well microtitre plates (Maxisorp) were from Nunc (Roskilde, Denmark).

### Monoclonal antibodies

Monoclonal antibodies (mAbs) were raised against recombinant Hc BoNT/A1 as previously described [20]. The relative epitopes of these mAbs and their subsequent classification in five epitopic groups were determined using complementary tests. The mAbs selected for their neutralizing activity were affinity-purified on protein A (Prosep-A High capacity, Millipore, Billerica, USA) and the F(ab')_2_ fragments obtained by treatment with pepsin in acidic medium [21]. Purity of mAbs and F(ab')_2_ was assessed by polyacrylamide gel electrophoresis (SDS-PAGE) in denaturing and reducing conditions using the phast System apparatus (GE Healthcare, Bucks, UK) with 10–15% polyacrylamide gels, following the manufacturer's instructions.

### F(ab')_2_ pegylation

After preparation, the F(ab')_2_ were dialyzed against borate buffer 0.02 M pH 9 before conjugation to PEG using a 3-fold molar excess of PEG (15 nmoles) added to 500 µg (500 µl of a 1 mg/ml solution) of dialyzed F(ab')_2_ (5 nmoles). After 30 min at room temperature with gentle agitation, F(ab')_2_ were purified by cation exchange chromatography to remove unreacted PEG and to separate the different F(ab')_2_ species (nonpegylated, monopegylated and multipegylated F(ab')_2_). A MacroCap SP column (GE Healthcare) (10.5×1 cm) was used with an AKTÄ system (GE Healthcare) and equilibrated with buffer A (0.02 M acetate buffer pH 5) at a flow rate of 1 ml/min. The reaction mixture was filtered through a 0.2 µm filter and loaded onto the column. After washing the column with 17 ml of buffer A at 1 ml/min, the pegylated F(ab')_2_ was eluted using successive gradient steps of 12 ml each at 2, 3, 4, 5 and 25% of buffer B (0.02 M acetate buffer pH 5+1 M NaCl) at 1 ml/min. The column was regenerated with 17 ml of buffer B at 1 ml/min. During the chromatography, fractions of 1 ml were collected and the absorbance was monitored at 280 nm. Fractions corresponding to each peak were pooled and analyzed by SDS-PAGE.

### Monoclonal antibody complementary tests and epitope mapping

The simultaneous binding of the different mAbs to BoNT/A was analyzed in immunometric tests, with one mAb immobilized on the solid phase while the other was used as a biotin-labeled tracer. Experiments were performed as follows: 10 ng/ml BoNT/A and 100 µl of a 100 ng/ml biotin-labeled mAb solution were added to microtiter plate wells coated with one of the mAbs. After 18-h incubation at 4°C, the plates were washed before adding 200 µl/well of AChE-labeled streptavidin conjugate (2 EU/ml). After a further 1-h incubation at room temperature followed by several washings, 200 µl of Ellman's medium were added to each well. After 1-h incubation, the absorbance was measured at 414 nm (Table S1).

For each pair of mAbs two combinations are possible. The absence of specific signals for both combinations means that these mAbs have the same or close epitopes.

### Enzyme immunoassay of anti-BoNT/A1 mAbs and their fragments in mouse plasma

All experiments were performed in accordance with French and European Community guidelines for laboratory animal handling. The pharmacokinetic studies were performed on mice using mouse monoclonal antibodies. The specific measurement of anti-BoNT/A1 immunoglobulins requires development of an original assay based on the recognition of BoNT/A1 by these mAbs or their fragments. In a previous study [20] we tested different combinations of anti-BoNT/A1 mAbs to design an immunometric assay. Among the pairs of mAbs able to bind BoNT/A1 simultaneously, some were tracer mAbs that recognized the same epitope as mAbs tested in the pharmacokinetic study (TA12 and TA17). When the sandwich assay was performed in the presence of a tracer mAb and another unlabeled mAb, both directed against the same epitope, there was competitive binding to the recombinant Hc BoNT/A1 immobilized on the plate via the capture mAb. This reaction led to a signal decrease proportional to the competitor concentration. To perform this assay, 50 µl of recombinant Hc BoNT/A1 (2 ng/ml) were added to 50 µl of mAb-AChE (2 EU/ml) and 100 µl of standard or samples, in plates coated with the complementary mAb. For the TA12 quantification, we used TA5 as capture antibody and TA12-AChE as tracer, while for TA17 we used TA16 as capture antibody and TA13 as tracer. After 18-h incubation at 4°C and washing of the plate, solid-phase bound AChE activity was revealed by the addition of 200 µl of Ellman's reagent for a 1-h reaction. The absorbance of the wells was then measured at 414 nm.

For accurate quantification, and depending on the experiments, the different forms of antibody (mAb, F(ab')_2_ or PEG-F(ab')_2_) injected in the mice were used as standard (Figure S1). The plasma samples were diluted in 20-fold in EIA buffer and the standards were prepared in plasma diluted 20-fold in EIA buffer. Higher sample dilutions were made by dilution of diluted sample (1/20) in diluted plasma (1/20). Each standard was analyzed in duplicate and the blank (buffer alone) in octuplicate in order to determine the minimum detectable concentration.

### Pharmacokinetic studies

Balb\c mice (male, 30 g) were from the Centre d'élevage René Janvier (Le Genest Saint Isle, France). An equivalent amount (0.33 nmoles) of mAbs, F(ab)'_2_ or PEG-F(ab')_2_ was injected in a single intraperitoneal dose. For each post-dose time, blood samples were collected from four animals by retro-orbital bleeding. Animals that received mAbs were sampled at the following post-dose times: 10 min, 4, 24, 48, 96, 240, 504 and 960 h. Animals that received F(ab')_2_ fragments were sampled at the following post-dose times: 10, 30 min and 1, 2, 4, 8, 18 and 24 h. Animals that received PEG-F(ab')_2_ were sampled at the following post-dose times: 30 min and 1, 4, 8, 18, 24, 48, 72, 96 and 240 h. Plasma samples were assayed for mAb, Fab'_2_ or PEG-Fab'_2_ fragments using the competitive sandwich procedure previously described. A noncompartmental pharmacokinetic analysis was performed on concentration-time data using the WinNonlin program.

### Evaluation of mAb (ascitic fluid) neutralizing activity

The neutralizing capacity of the antibodies was evaluated in the *in vivo* mouse lethality test. Neutralization tests were performed using acid-precipitated toxins from strain Hall (type A1). Ten-fold serial dilutions of the fourteen mAbs were incubated with botulinum toxin type A1 containing approximately 10 mouse 50% lethal doses (MLD_50_/ml) (Table S2), in 50 mM phosphate buffer pH 6.5 containing 1% gelatin (PB-G), for 30 min at room temperature. The mixtures (0.5 ml) were then injected intraperitoneally (i.p.) into Swiss mice weighing 20–22 g (4 mice per group). Mice were observed and any deaths recorded every day for 4 days.

### Neutralizing activity of TA12, TA17 and their fragments

The neutralizing capacity of the antibodies was evaluated in the *in vivo* mouse lethality test. Neutralization tests were performed using acid-precipitated toxins from strain Hall (type A1), strain 136.06 (type A2; GenBank HM135956) and strain Loch Maree (type A3). Ten-fold serial dilutions of TA12, TA17 mAbs or their fragments were incubated with botulinum toxin type A1, A2 or A3 containing approximately 10 mouse 50% lethal doses (MLD_50_/ml) (Table S2), in PB-G, for 30 min at room temperature. The mixtures (0.5 ml) were then injected i.p. into Swiss mice weighing 20–22 g. Groups of 6–12 mice were used in three independent assays. Mice were observed and any deaths recorded every day for 4 days.

### Neutralizing activity using co-injection protocol

The purpose of this experiment was to determine the neutralizing activity of TA12 mAb and its F(ab')_2_ fragments, before and after pegylation, co-injected with the toxin without preincubation.

The neutralizing capacity of the antibodies was evaluated in the *in vivo* mouse lethality test. 5 estimated MLD50 of BoNT/A1 in PB-G (0.5 ml) were injected i.p. into Swiss mice weighing 20–22 g (6 mice per group). Concomitantly, 0.5 ml of various dilutions of mAbs or their fragments in PB-G was injected i.p. into a close but different site. Mice were observed and any deaths recorded every day for 4 days.

### Time-course of clinical signs

5 estimated MDL_50_ of BoNT/A1 were injected i.p. into 8 Swiss mice and the clinical signs of botulism were then recorded every half hour for 8 hours.

### Influence of the interval between toxin and anti-toxin injections

Four groups of 17 to 18 mice for TA12 mAb and 4 groups of 10 mice for the F(ab')_2_ and PEG-F(ab')_2_ fragments were used. At T = 0, 5 estimated MDL_50_ of BoNT/A1 were injected i.p. into each mouse. The antitoxin was then injected i.p. after 0, 2, 4 or 6 hours, depending on the group of mice. For the experiment, we used antitoxin equivalent to 100 times the dose which protected 100% of the mice challenged with 5 estimated MLD50 in the co-injection experiment. Botulism symptoms and deaths were recorded for 4 days.

## Results

### Measurement of BoNT/A1 neutralizing activity

In a previous study [20] using a monoclonal antibody complementary test (Table S1), we classified the 14 mAbs produced against the Hc BoNT/A1 protein into 5 distinct epitopic groups. Groups A, B and C contain 5 mAbs (TA2, TA10, TA11, TA12 and TA14), 4 mAbs (TA4, TA7, TA13 and TA17) and 3 mAbs (TA1, TA5, TA15), respectively, while the groups D and E include a single mAb only (TA9 and TA16, respectively). As shown in Table 1, ascitic fluids from most of these mAbs (12/14) have neutralizing activity. It is noteworthy that although all the mAbs recognized BoNT/A1 by ELISA, the mAbs of group C exhibited very low or even no neutralizing activity. As the immunoglobulin (Ig) content of ascitic fluids may vary from 1 to 10 mg/ml, we repeated the same experiment with the most potent mAb of each group after purification to compare more precisely the neutralization activity of the different mAbs (Table 2).

**Table 1:** BoNT/A1 neutralization activity of crude ascite fluids from 14 mAbs in the mouse protection assay (5 estimated MLD_50_/mouse).

Epitopic group	Antibody	Isotype	Protective titer
**A**	TA2	IgG1	10^4^
	TA10	IgG1	10^3^
	TA11	IgG1	10^4^
	**TA12**	**IgG1**	**10^5^**
	TA14	IgG1	10^4^
**B**	TA4	IgG2a	10^4^
	TA7	IgG2a	10^4^
	TA13	IgG2a	10^3^
	**TA17**	**IgG2a**	**10^4^**
**C**	TA1	IgG1	≤**10^2^**
	TA5	IgG1	≤**10^2^**
	TA15	IgG1	≤**10^2^**
**D**	**TA9**	**IgG1**	**10^2^**
**E**	**TA16**	**IgG1**	**10^3^**

The protective titer is the highest dilution of the ascitic fluid that completely protected against 5 estimated MLD_50_ of BoNT/A1 in the mouse protection assay.

Underline: non-neutralizing antibodies. Three ml of 10 estimated MLD_50_/ml BoNT/A1 were incubated with 30 µl of serial ten-fold dilutions of ascitic fluid for 30 min at room temperature. 500 µl of the mixtures were injected intraperitoneally to give final toxin challenges of approximately 5 MLD/_50_ into each mouse of a group of 4 for each dilution.

**Table 2:** BoNT/A1 neutralization activity of four purified monoclonal antibodies.

Epitopic group	Antibody	Ig concentration (mg/ml)	Protective titer	Lowest protective amount*
A	TA12	0.442	10^5^	2.2 ng
B	TA17	1.4	10^3^	700 ng
D	TA9	0.595	10^2^	∼3 µg
E	TA16	0.608	10^2^	∼3 µg

The protective titer is the highest dilution of the purified antibody that allows the neutralization of 5 estimated MLD_50_ of BoNT/A1 neurotoxin in the mouse protection assay.

Ten-fold dilutions were used to generate final antibody concentrations ranging from 2.21 ng-2.21 µg/mouse for TA12, 700 ng-7 µg for TA17, 5.9 µg for TA9, and 6.1 µg for TA16. These dilutions were incubated with toxin for 30 min and 0.5 ml was injected to give final toxin challenges of approximately 5 MLD_50_/mouse of a group of 4 for each dilution.

*Lowest amount of antibody needed to completely protect the mice against a 5 estimated MLD_50_ toxin.

**Figure 1:**
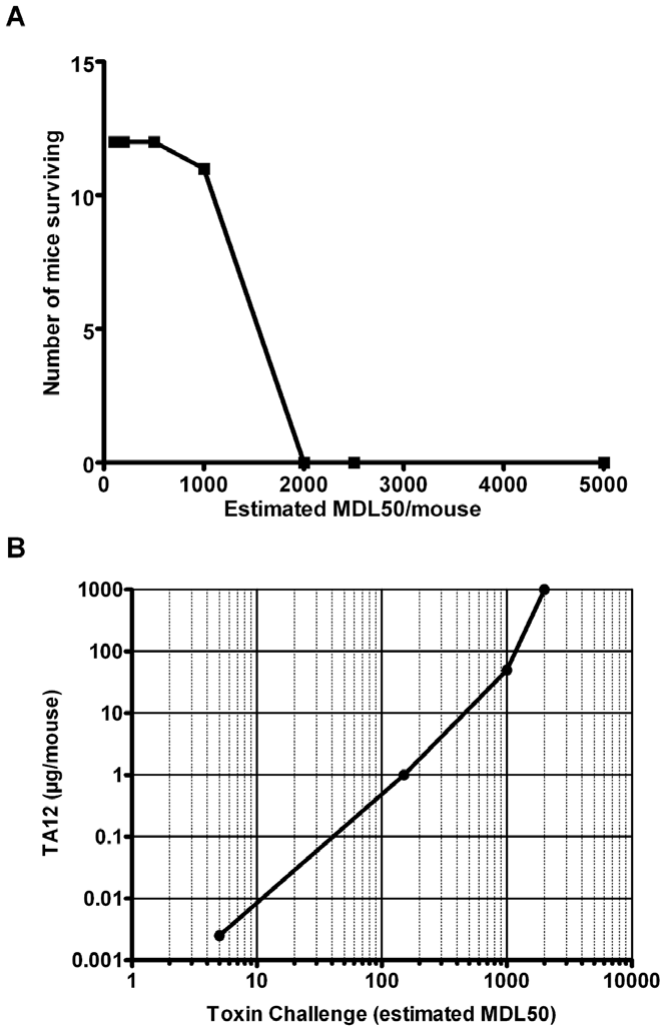
BoNT/A1 neutralization activity of TA12 mAb. **A.** TA12 mAb (50 µg/mouse) was incubated with variable amounts of BoNT/A1 from 100 to 5,000 estimated MLD_50_/mouse and the mixtures were intraperitoneally injected to groups of 12 mice each. The number of mice surviving versus challenge is indicated. TA12 mAb (50 µg) protected approximately 1,000 estimated MLD_50_. **B**. Protective doses of TA12 mAb with increasing BoNT/A1 challenge doses. Protective potency of various doses of TA12 mAb (0.0025, 1, 50, 1,000 µg/mouse) was determined by incubation with various BoNT/A1 challenge doses. The mixtures were injected intraperitoneally to groups of 12 mice. The protective TA12 mAb doses protecting 90 to 100% mice are plotted versus BoNT/A1 challenge doses. Note that increasing amounts of TA12 mAb are required to neutralize increased BoNT/A1 challenge doses, specially above 1,000 estimated MLD_50_.

This experiment emphasized the great potency of TA12 mAb in neutralizing BoNT/A1 toxicity in mice, since 2.2 ng of antibody completely protected mice against an 5 estimated LD50 challenge of BoNT/A1. In order to compare the protective activity of TA12 mAb with other recent reported mAbs, we also studied the ability of a fixed amount of TA12 antibody (50 µg/mouse) to protect mice challenged with increasing amount of BoNT/A1 ranging from 100–10,000 MLD_50_ (Figure 1A). In this experiment, TA12 mAb showed a significant in vivo protection when mice were challenged with 1,000 MLD_50_. As combinations of several mAbs neutralize more efficiently than individual mAbs [11], we tested different combinations of TA12 mAb with other neutralizing mAbs of other groups. We failed to find a higher neutralization titer than that obtained using TA12 mAb alone. TA12 was also titrated with the reference L+10 method (European Pharmacopoeia 01/2008:0085) and international standard antitoxin A (NIBSC 59/21) and a titer of 6 to 20 IU/mg of antibody was obtained (by definition, an International Unit (IU) neutralizes 1×10^4^ MLD_50_). Again, in order to compare the protective activity with other reported mAbs, the potency of TA12 was also titrated using the standard mouse bioassay described by Hatheway and Dang [22] and was determined to be 286 UI/mg of Ab. Since it has been found that the neutralization activity of anti-BoNT/A antibodies is not linear using various challenge doses, the protective activity of TA12 was determined with several BoNT/A1 doses (Figure 1B). As the toxin challenge increases, the level of TA12 needed to completely protect mice also increased and appeared to reach a maximum at approximately above 50 µg of antibody and 1,000 MLD_50_ (Figure 1B). This experiment also showed that the protective activity of TA12 is not linear over the range of 5–2,000 MLD_50_. For further studies, 2 mAbs were selected: TA12, since it has the highest BonT/A1 neutralizing activity, and TA17, which is quite efficient and recognizes a different epitope than TA12.

### BoNT/A2 and/A3 neutralizing activity of TA12 and TA17

As currently defined, BoNT/A consists of various subtypes [23]–[25]. Amino acid sequence variations within BoNT/A subtypes are around 15% or less and have been reported to impact on antibody binding and neutralization [26]. We therefore studied the capacity of the purified TA12 and TA17 mAbs to neutralize subtypes other than A1, such as BoNT/A2 and BoNT/A3. As depicted in Table 3, TA12 and TA17 mAbs showed a marked (10- to 100-fold) reduction in capacity to neutralize BoNT/A2 and BoNT/A3, compared with BoNT/A1.

**Table 3:** Neutralization activity of TA12 and TA17 mAbs with BoNT/A subtypes.

mAb quantity (ng/mouse)		2,500	250	25	2.5	0.25	mAb quantity (ng/mouse) giving 50% neutralization activity
**TA12**	BoNT/A1	0/10	0/10	0/10	0/10	10/10	0.25<5
	BoNT/A2	0/9	6/9	9/9	ND	ND	250
	BoNT/A3	0/9	0/9	6/9	ND	ND	25
**TA17**	BoNT/A1	0/10	0/10	10/10	ND	ND	25<250
	BoNT/A2	3/9	10/10	10/10	ND	ND	2 500
	BoNT/A3	10/10	10/10	8/10	ND	ND	>2 500

ND: not determined

MAb neutralization activity was determined using the mouse protection assay with 5 estimated MLD_50_/ml of BoNT/A1, BoNT/A2 or BoNT/A3 and serial dilutions of mAbs.

5 estimated MLD_50_/mouse were incubated for 30 min at room temperature with 2,500 to 0.25 ng of mAb and the mixture (0.5 ml) was injected intraperitoneally in each mouse of a group of 9 to 10 mice.

Results are expressed as the number of dead mice versus the total number of mice and are from three independent experiments.

### F(ab')2 pegylation

**Figure 2:**
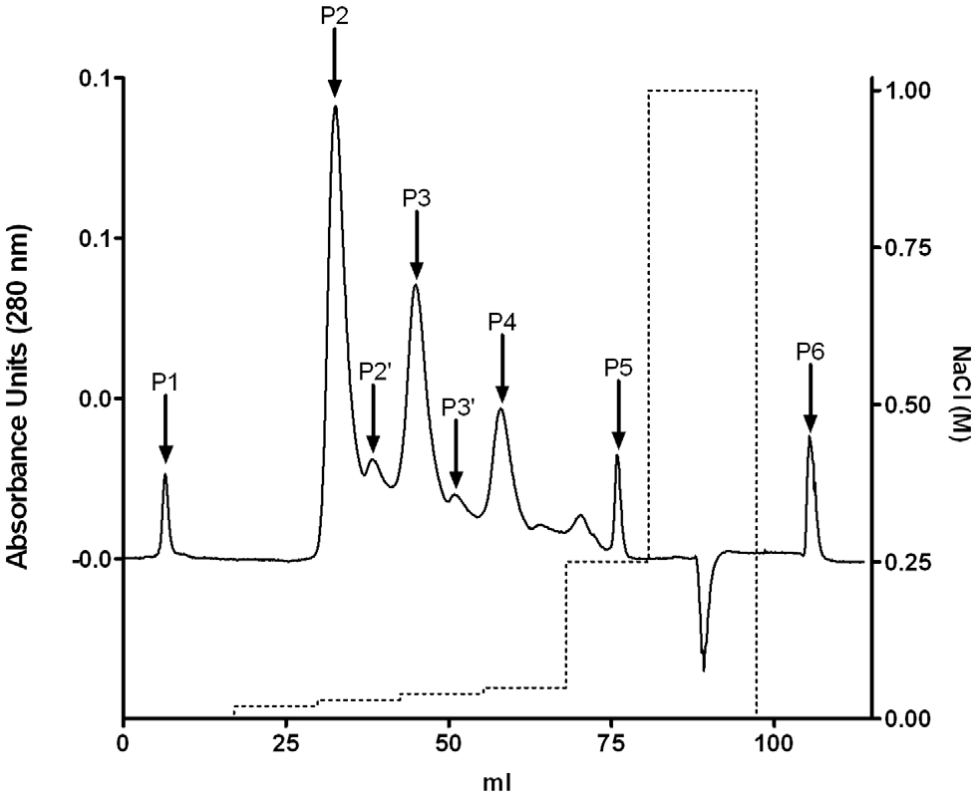
Elution profile of PEG-F(ab')_2_. — UV detection at 280 nm; ----- NaCl concentration; P1, P2, P2′, P3, P3′, P4, P5 and P6 correspond to the different peaks collected during the chromatography purification. After conjugation of F(ab')_2_ to the N-hydroxysuccinimidyl ester of methoxy poly(ethylene glycol) butanoic acid (NHS-PEG), the different F(ab')_2_ species were purified by cation exchange chromatography. This purification removed unreacted NHS-PEG and separated the different F(ab')_2_ species (nonpegylated, monopegylated and multipegylated F(ab')_2_). A MacroCap SP column (GE Healthcare), (10.5×1 cm) was used with an AKTÄ system (GE Healthcare) and equilibrated with buffer A (0.02 M acetate buffer pH 5) at a flow rate of 1 ml/min. The pegylated F(ab')_2_ were eluted using successive gradient steps of 12 ml each at 2, 3, 4, 5 and 25% of buffer B (0.02 M acetate buffer pH 5 + 1 M NaCl) at a flow rate of 1 ml/min. During the chromatography, fractions of 1 ml were collected and the absorbance was monitored at 280 nm.

**Figure 3:**
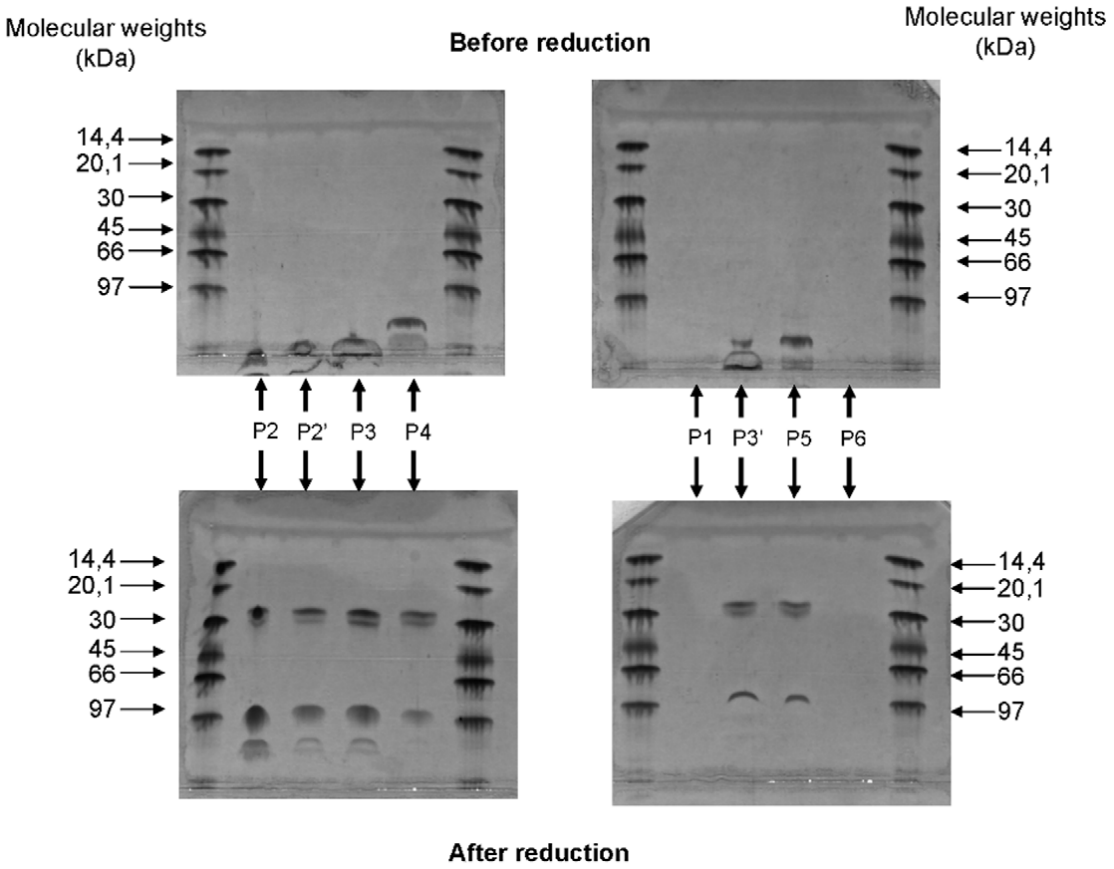
SDS-PAGE analysis of the PEG-F(ab')_2_ chromatography peaks. P1, P2, P2′, P3, P3′, P4, P5 and P6 correspond to the different peaks recorded during the chromatography purification. KDa: KiloDalton After conjugation of F(ab')_2_ to the N-hydroxysuccinimidyl ester of methoxy poly(ethylene glycol) butanoic acid (NHS-PEG), the different F(ab')_2_ species were purified by cation exchange chromatography. This purification removed unreacted NHS-PEG and separated the different F(ab')_2_ species (nonpegylated, monopegylated and multipegylated F(ab')_2_).

The protocol used for the pegylation process was quite simple, but resulted in several species of PEG-F(ab')_2_ (one or several PEG molecules per F(ab')_2_) and no native F(ab')_2_, as revealed by SDS-PAGE electrophoresis (data not shown). In order to purify each PEG-F(ab')_2_ species, we used a cation exchange matrix, allowing the purification of large pegylated molecules. The chromatographic pattern of the PEG-F(ab')_2_ purification monitored at 208 nm is shown in Figure 2. The purity of each peak was assessed by SDS-PAGE (Figure 3). As shown in Figure 3, a good separation of PEG-F(ab')_2_ was obtained using a multi-step gradient; no native F(ab')_2_ was recovered. Since a rather low molar ratio of PEG activated ester was used, this result is a bit surprising. During the chromatographic experiment the different PEG-F(ab')_2_ species were eluted depending on their degree of pegylation: the more pegylated species were eluted faster. As indicated in the introduction section, production of PEG-F(ab')_2_ was chosen because this process easily increases the (Fab)'_2_ *in vivo* half-life. As the aim of this production was to increase the *in vivo* half-life of the F(ab')_2_ fragments and not to study the effect of the degree of pegylation, which has already been investigated by other groups, the different fractions containing PEG-F(ab')_2_ were pooled for the subsequent experiments. This allowed us to obtain a larger amount of PEG-F(ab')_2_ starting from the same amount of F(ab')_2_.

### Pharmacokinetic studies

**Figure 4:**
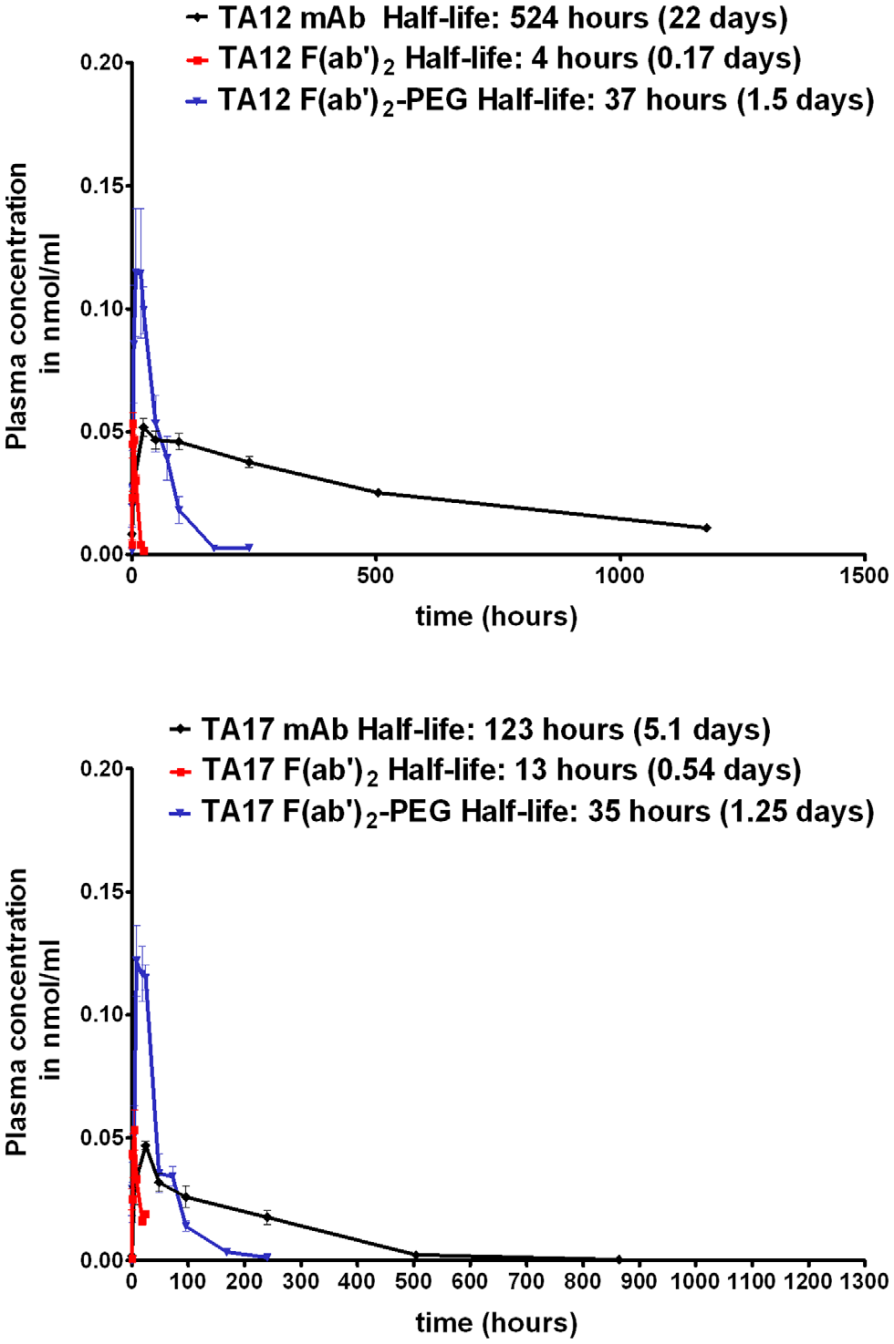
Mean concentration-time profiles following administration of 0.33 nmoles of mAb, F(ab')_2_ or PEG-F(ab')_2_. An equivalent amount (0.33 nmoles) of mAbs, F(ab)'_2_ or PEG-F(ab')_2_ was injected in Balb\c mice in a single intraperitoneal dose. For each post-dose time, blood samples were collected from four animals by retro-orbital bleeding. Animals that received mAbs were sampled at the following post-dose times: 10 min, 4, 24, 48, 96, 240, 504 and 960 h. Animals that received F(ab')_2_ fragments were sampled at the following post-dose times: 10, 30 min and 1, 2, 4, 8, 18 and 24 h. Animals that received PEG-F(ab')_2_ were sampled at the following post-dose times: 30 min and 1, 4, 8, 18, 24, 48, 72, 96 and 240 h. Plasma samples were assayed for mAb, Fab'_2_ or PEG-Fab'_2_ fragments.

The concentrations of mAb, F(ab')_2_ and PEG-F(ab')_2_ in the plasma of mice (n = 4) versus time are depicted in Figure 4. The calculated half-lives in plasma for TA12 mAb, TA12 F(ab')_2_, TA12 PEG-F(ab')_2_, TA17 mAb, TA17 F(ab')2 and TA17 PEG-F(ab')_2_ were 22, 0.17, 1.5, 5.1, 0.54 and 1.25 days, respectively. These results clearly show that the pegylation increased the half-life of the F(ab')_2_ fragments. Compared with unmodified F(ab')_2_, the terminal half-life of PEG-F(ab')_2_ was increased 9- and 3-fold for TA12 and TA17, respectively.

### Neutralizing activity of the mAbs and their fragments

The BoNT/A1-neutralizing activities of mAbs and their fragments are summarized in Tables 4 and 5. Unlike mAb TA12, which had the same neutralizing titer whatever its form, the neutralizing titer of the TA17 antibody decreased 10-fold when using F(ab')_2_ fragments, pegylated or not. It is worth noting that the pegylation step did not modify the neutralization capacity of either of these antibodies. On the other hand, TA12 mAb and its fragments had a much greater neutralizing activity than TA17 mAb and its fragments: 100-fold for the whole antibody and 1,000-fold for the fragments. Therefore, we decided to study the therapeutic activity of TA12 mAb and its fragments.

**Table 4:** Neutralizing potency of the TA12 mAb and its F(ab')_2_ and PEG- F(ab')_2_ fragments.

Antibody and antibody fragment		mAb (ng/mouse)						mAb (ng/mouse) giving 50% neutralization activity
		2,500	250	25	2.5	0.25	0.025	
TA12	mAb	0/10	0/10	0/10	4/10	6/8	6/8	2.5
	F(ab')2	0/10	0/10	2/10	3/9	8/8	8/8	2.5
	PEG-F(ab')2	0/10	0/10	0/10	2/8	6/8	8/8	0.25<2.5

The mAb neutralizing activity was determined using the mouse protection assay with 5 estimated MLD_50_/mouse of BoNT/A1.

5 estimated MLD_50_/mouse were incubated for 30 min at room temperature with 2,500 to 0.025 ng of mAb and the mixture (0.5 ml) was injected intraperitoneally in each mouse of a group of 8 mice.

Results are expressed as the number of dead mice versus the total number of challenged mice and are from three independent experiments

**Table 5:** Neutralizing potency of the TA17 mAb and its F(ab')_2_ and PEG- F(ab')_2_ fragments.

Antibody and antibody fragment		mAb (ng/mouse)					mAb (ng/mouse) giving 50% neutralization activity
		8,333	2,500	500	250	50	
TA17	mAb	0/8	0/8	0/8	0/8	8/8	50<250
	F(ab')_2_	0/8	8/8	6/8	8/8	ND	2,500<8,333
	PEG-F(ab')_2_	0/8	4/8	6/8	6/8	ND	2,500<8,333

ND: not determined

The mAb neutralizing activity was determined using the mouse protection assay with 5 estimated MLD_50_/mouse of BoNT/A1.

5 estimated MLD_50_/mouse were incubated for 30 min at room temperature with 8,333 to 50 ng of mAb and the mixture (0.5 ml) was injected intraperitoneally in each mouse of a group of 8 mice.

Results are expressed as the number of dead mice versus the total number of challenged mice and are from three independent experiments.

### Therapeutic Study

#### Neutralizing activity using co-injection

As shown in Table 6, the amount of antibody required to neutralize 5 estimated MDL_50_ of BoNT/A1 was 10 times higher when the toxin was co-injected with the antibody than when seroneutralization is performed *in vitro* before injection of the mixture (25 ng of mAb/mouse and 2.5 ng of mAb/mouse, respectively). This observation is true whatever the antibody forms (mAb, F(ab')_2_ or PEG-F(ab')_2_).

**Table 6:** Neutralizing potency of the mAb TA12 and its F(ab')_2_ and PEG-F(ab')_2_ fragments using the co-injection protocol.

TA12 form	mAb (ng/mouse)						mAb (ng/mouse) giving 50% neutralization activity
	2,500	250	25	2.5	0.25	0.025	
mAb	0/6	2/6	1/6	4/6	3/6	6/6	2.5<25
F(ab')_2_	ND	ND	0/6	3/6	6/6	6/6	2.5<25
PEG-F(ab')_2_	ND	ND	0/6	3/6	3/6	6/6	2.5<25

ND: not determined

The mAb neutralizing activity was determined using the mouse protection assay with 5 estimated MLD_50_/mouse of BoNT/A1. BoNT/A1 (0.5 ml) and serial dilutions of mAb or its fragments (0.5 ml) were injected separately into each mouse of a group of 6. Results are expressed as the number of dead mice versus the total number of challenged mice.

This result seems consistent with the preincubation step used during the standard bioassay which favors the formation of toxin/antitoxin complexes, and thus the efficiency of neutralization.

#### Time course of clinical signs

The time course of the onset of clinical signs varied according to the mice (Table 7). The first clinical signs (skinny or paralyzed mouse) appeared after 4 to 8 hours. The death of the first mouse was observed after 7 hours (1/7). All the mice presenting clinical signs died within 24 hours.

**Table 7:** Time course of clinical signs after botulinum toxin type A injection (5 estimated MLD_50_/mouse).

Mouse #	Before 4 h	4 h	5 h	5.5 h	6 h	6.5 h	7 h	7.5 h	8 h	24 h
1	No sign	A	A	A	A	A	A	A	A	A
2	No sign	S	S	P	P	P	P	P	M	D
3	No sign	S	S	P	P	P	P	M	M	D
4	No sign	S	S	S	S	S	S	S	P	D
5	No sign	S	S	P	P	P	P	M	M	D
6	No sign	P	P	P	M	M	D	D	D	D
7	No sign	S	P	P	P	P	M	M	M	D
8	No sign	S	S	S	P	P	P	P	M	D

A: Alive; S: Skinny; P: Paralyzed; M: Motionless; D: Dead

The indicated times correspond to the time after botulinum toxin injection

#### Influence of the interval between toxin and antitoxin injections on mouse survival rate

Mouse survival rate as a function of the interval between toxin and antitoxins is summarized in Table 8. When 5 estimated MDL_50_ of BoNT were injected i.p. at the same time as 2.5 µg of antitoxin, the survival rate was approximately 90%, whatever the nature of the antitoxin (whole mAb, F(ab')_2_ or PEG-F(ab')_2_). This rate decreased as the time between toxin and antitoxin injections increased: 70% for 2 h, 20 to 40% for 4 or 6 h. It should be noted that for an interval of 6 hours, when the clinical signs were well established, 30 to 40% of the animals survived and recovered gradually without additional treatment.

**Table 8:** Influence of the interval between toxin and antitoxin injections on survival rate (values in brackets).

Antitoxin form	0 hours	2 hours	4 hours	6 hours
TA12 mAb	16/17 (94%)	12/18 (67%)	9/18 (50%)	5/18 (28%)
TA12 F(ab')_2_	9/10 (90%)	8/10 (80%)	4/10 (40%)	4/10 (40%)
TA12 PEG-F(ab')_2_	9/10 (90%)	7/10 (70%)	2/10 (20%)	4/10 (40%)

The indicated times correspond to the interval between toxin and antitoxin injections.

At T = 0 5 estimated MDL_50_ of botulinum toxin type A1 (0.5 ml) were injected i.p. into all mice. The antitoxin was then injected i.p. into mice after 0, 2, 4 or 6 hours. An amount of antitoxin (2.5 µg/mouse) equivalent to 100 times the dose which protected 100% of the mice challenged with 5 estimated MLD50 in the co-injection experiment was used. Botulism symptoms and mortality were recorded for 4 days. Results are expressed as the number of dead mice versus the total number of challenged mice.

## Discussion

As previously shown by different groups [19], [27], [28], the use of the BoNT binding domain as immunogen allows production of a high ratio of neutralizing mAbs. In this study, 12 out of 14 mAbs presented neutralizing activity and recognized 4 different epitopes of the toxin-binding domain, which was used as antigen. Among these mAbs, TA12 had the highest neutralizing activity (Table 1). In contrast to the results reported by Nowakowski et al. [11], who reported increased neutralization efficiency for a combination of three mAbs, none of the combinations of TA12 with other neutralizing mAbs were more potent. This may be because of the already high neutralizing capacity of TA12 (6 to 20 IU/mg using the reference L+/10 method or 286 IU/mg by the method from Hatheway and Dang [22]), which alone is as efficient, or slightly less so, as other antibodies reported in the literature, such as an oligoclonal antibody (45 IU/mg, [11]), a hyperimmune mono-serotype horse type antitoxin (39 IU/mg [29]), and a caprine pentavalent polyclonal (6.8 IU/mg, [5]). TA12 is also more potent than the human botulinum immune globulins used to treat infant botulism [30]. To the best of our knowledge, TA12 exhibits the highest neutralization potency described for a single mAb as a BoNT/A1 antitoxin. However, the mouse lethal bioassay could be influenced by factors like season, strain of mouse, diet, toxin dilution buffer, bacterial strain producing the toxin, toxin challenge dose and route of injection [5]. So the comparison between the potency of different antitoxins obtained by different groups is questionable.

Recently, variability of BoNT/A protein sequence has been reported and at least 5 subtypes have been identified: BoNT/A1/A2/A3/A4/A5 [31] [25]. A recent report [26] has shown that the variability within subtypes A1 and A2 (around 10%) could reduce the neutralizing potency of mAbs raised against recombinant Hc BoNT/A1, from >40, 000 mouse MLD_50_ to 500 MLD_50_. In light of these observations, we decided to evaluate the neutralizing potency of TA12 mAb with BoNT/A2 and BoNT/A3. The neutralizing activities obtained were 10- and 1,000-fold less for BoNT/A2 and A3 subtypes, respectively, compared with BoNT/A1 (Table 3). Combinations of our mAbs may possibly increase the potency of neutralization of BoNT/A2 and BoNT/A3, as already described [11]. It is worth noting that the neutralization assays were performed with a 5 estimated MLD_50_ challenge of each BoNT subtype, since no international reference serum is available for titration of BoNT/A2 and BoNT/A3 antibodies by the reference L+10 method.

The main goal of this study was to evaluate the impact of the different antitoxin forms (whole antibody, F(ab')_2_ and PEG-F(ab')_2_) on their neutralizing efficiency. The procedures used to modify the F(ab')_2_ lead to several species of PEG-F(ab')_2_ with different degrees of pegylation. The pharmacokinetic study of these antitoxins showed that the pegylation process increased the half-life of F(ab')_2_ for both antibodies evaluated. Although the molecular weight of F(ab')_2_—98 k Da—is already above the limit of the glomerular filtration cut-off (approximately 70 kDa), the increased half-life of pegylated products may be partly explained by an increase in molecular weight, further resulting in a decrease in urinary clearance. This may be supported by the results reported by Knauf et al [32] and Koumenis et al. [14] showing a relationship between the clearance rate and the molecular weight of pegylated molecules up to 200 kDa and 1.9 million Da, respectively. This relationship could also be explained by several other mechanisms, like shape alteration and protein charge induced by pegylation. On the other hand, whole immunoglobulins G have a higher half-life probably because their clearance is slower than that of their respective fragments. This is probably due to specific Hc interactions with receptors like HcRn which protect IgG from catabolism [33], [34].

By contrast with TA17 mAb, the production of F(ab')_2_ fragment from TA12 mAb by pepsinolysis did not modify the neutralizing activity. Moreover, as reported by Koumenis et al. [14], the random pegylation of the F(ab')_2_ fragments of both mAbs by amine reactive chemistry did not affect the biological activity. Considering these results, the therapeutic study was performed only with the TA12 mAb and its fragments, since these compounds were at least 100 times more efficient than the TA17 counterparts.

The time course of the onset of clinical signs induced by BoNT/A injection defines the optimal timing of antitoxin injection and the clinical course after treatment. Using the data of co-injection experiments, the impact of the interval between toxin and antitoxin injections on the survival rate was evaluated. This study showed that the various forms of TA12 mAb were equally efficient. Amazingly, the 4-h interval using PEG-F(ab')_2_ resulted in a poorer survival rate, which is difficult to explain considering the data for the 6-h interval and may result from an experimental artifact. These results strongly suggest an absence of correlation between the half-life of the antitoxins and the timing of treatment after contamination. This seems consistent with the time required for the toxin and antitoxin to reach their action sites (neuromuscular junctions for the toxin, blood and neuromuscular junctions for the antitoxin) and with the mode of action of the antitoxin. In our study, TA12 mAb, which has been raised with Hc BoNT/A1, or its fragments presumably neutralized BoNT by preventing its binding to the cellular membrane receptors (gangliosides and proteins), the step preceding its internalization. Internalization further prevents the toxin from interacting with mAbs, which can therefore only act efficiently during the initial step, i.e. when the toxin is free, by blocking toxin binding to its receptors. It is noteworthy that in human intoxication by the oral route or aerosol the incubation period is longer (12 h to 7 days) than in the murine experimental model and, moreover, the amount of ingested or inhaled toxin can be lower than the lethal dose. In these cases, the administration of antibody to exposed subjects is still indicated to prevent botulism effectively. However, when the clinical signs are present, the time scale allowing effective action of botulinum antibody antitoxin is very short, if it is not already too late.

Despite these results, since the biological activity of mAbs could be altered by the F(ab')_2_ production, as already observed with TA17 mAb in the present study, or by F(ab')_2_ pegylation [17], the production of chimeric or humanized antibodies is still a good alternative to production of human anti-mouse antibodies. In terms of the prophylactic use of these antitoxins, it should be kept in mind that their half-life is the key parameter for the duration of protection.

In conclusion, the present study shows that while pegylation increases the half-life of F(ab')_2_ without altering its neutralizing efficiency, it does not improve therapeutic treatment after BoNT/A1 injection, as compared with the initial F(ab')_2_ or mAb. Moreover, our results show that, in a mouse model, treatment can still be effective with a 2- to 4-h interval between toxin and antitoxin injection.

Finally, these results demonstrate the need to make the diagnosis and to perform toxinotyping of botulism as fast as possible in order to expedite adaptation of the antitoxin treatment.

## Supporting Information

Figure S1 Examples of standard curves obtained for competitive sandwich assay of TA12 species (mAb, F(ab')2 or PEG-F(ab')2) in diluted plasma. ▪: TA12 mAb; ▾: TA12 F(ab')2; ▴: TA12 PEG-F(ab')2. The competitive sandwich assay is performed in the presence of a mAb tracer and another unlabeled mAb or fragment, both directed against the same epitope. There was competitive binding to the recombinant Hc BoNT/A1 immobilized on the plate by the capture mAb. This reaction leads to a signal decrease proportional to the competitor concentration. To perform this assay, 50 µl of recombinant Hc BoNT/A1 (2 ng/ml), 50 µl of TA12-acetylcholinesterase (AChE) and 100 µl of different concentrations of TA12 species (mAb, F(ab')2 or PEG-F(ab')2) were added to plates coated with TA5. The TA12 species were dilutions were made in diluted mouse plasma (1/20). After 18-h incubation at 4°C and washing of the plate, solid phase-bound AChE activity was revealed by the addition of 200 µl of Ellman's reagent for a 1-h reaction. The absorbances of the wells were then measured at 414 nm.
(0.81 MB TIF)

Table S1 Signals obtained for monoclonal antibody complementary tests. +++: Signals >1 absorbance unit; ++: Signals between 1 and 0.5 absorbance unit; +: Signals between 0.5 and 0.1 absorbance unit; +/−: Signals <0.1 absorbance unit; White squares: No specific signal. The simultaneous binding of the different mAbs to BoNT/A was analyzed in immunometric tests, with one mAb immobilized on the solid phase while the other was used tracer. Experiments were performed as follows: 10 ng/ml BoNT/A and 100 µl of a 100 ng/ml mAb tracer solution were added to microtitre plate wells coated with one of the mAbs. After 18-h incubation at 4°C, the plates were washed and the mAb tracer signal was measured (colorimetric signal).
(0.07 MB DOC)

Table S2 Toxin calibration. Toxin preparations were diluted in phosphate/gelatin buffer to obtain 5 estimated MLD/mouse and were checked as follows: 500 µl of toxin dilution were injected intraperitoneally into each mouse of a group of 3 to 4 mice. Results are expressed as the number of dead mice versus the total number of mice and are from 8 to 15 independent experiments.
(0.03 MB DOC)

## References

[1] Broussard LA (2001). Biological agents: weapons of warfare and bioterrorism.. Mol Diagn.

[2] Liu W, Montana V, Chapman ER, Mohideen U, Parpura V (2003). Botulinum toxin type B micromechanosensor.. Proc Natl Acad Sci U S A.

[3] Franz DR, Pitt LM, Clayton JM, Hanes MA, Rose KJ, DasGupta BR (1993). Efficacy of prophylactic and therapeutic administration of antitoxin for inhalation botulism.. Botulinum and Tetanus Neurotoxins: Neurotransmission and Biomedical Aspects.Plenum Press, N.Y.

[4] Wheeler MW (1923). Production of Monovalent botulinus antitoxic serum types A and B.. The Journal of Immunology.

[5] Jones RGA, Alsop TA, Hull R, Tierney R, Rigsby P (2006). Botulinum type A toxin neutralisation by specific IgG and its fragments: A comparison of mouse systemic toxicity and local flaccid paralysis assays.. Toxicon.

[6] Pless DD, Torres ER, Reinke EK, Bavari S (2001). High-affinity, protective antibodies to the binding domain of botulinum neurotoxin type A.. Infection and Immunity.

[7] Wu HC, Yen CT, Huang YL, Tarn LJ, Lung CC (2001). Characterization of neutralizing antibodies and identification of neutralizing epitope mimics on the Clostridium botulinum neurotoxin type A.. Applied and Environmental Microbiology.

[8] Torii Y, Tokumaru Y, Kawaguchi S, Izumi N, Maruyama S (2002). Production and immunogenic efficacy of botulinum tetravalent (A, B, E, F) toxoid.. Vaccine.

[9] Black RE, Gunn RA (1980). Hypersensitivity Reactions Associated with Botulinal Antitoxin.. American Journal of Medicine.

[10] Amersdorfer P, Wong C, Chen S, Smith T, Deshpande S (1997). Molecular characterization of murine humoral immune response to botulinum neurotoxin type A binding domain as assessed by using phage antibody libraries.. Infection and Immunity.

[11] Nowakowski A, Wang C, Powers DB, Amersdorfer P, Smith TJ (2002). Potent neutralization of botulinum neurotoxin by recombinant oligoclonal antibody.. Proceedings of the National Academy of Sciences of the United States of America.

[12] Smith TW, Lloyd BL, Spicer N, Haber E (1979). Immunogenicity and Kinetics of Distribution and Elimination of Sheep Digoxin-Specific Igg and Fab Fragments in the Rabbit and Baboon.. Clinical and Experimental Immunology.

[13] Mayers CN, Veall S, Bedford RJ, Holley JL (2003). Anti-immunoglobulin responses to IgG, F(ab')(2), and fab botulinum antitoxins in mice.. Immunopharmacology and Immunotoxicology.

[14] Koumenis IL, Shahrokh Z, Leong S, Hsei V, DeForge L (2000). Modulating pharmacokinetics of an anti-interleukin-8 F(ab')(2) by amine-specific PEGylation with preserved bioactivity.. International Journal of Pharmaceutics.

[15] Leong SR, DeForge L, Presta L, Gonzalez T, Fan A (2001). Adapting pharmacokinetic properties of a humanized anti-interleukin-8 antibody for therapeutic applications using site-specific pegylation.. Cytokine.

[16] Chapman AP, Antoniw P, Spitali M, West S, Stephens S (1999). Therapeutic antibody fragments with prolonged in vivo half-lives.. Nature Biotechnology.

[17] Chapman AP (2002). PEGylated antibodies and antibody fragments for improved therapy: a review.. Advanced Drug Delivery Reviews.

[18] Shone CC, Tranter HS (1995). Growth of clostridia and preparation of their neurotoxins..

[19] Tavallaie M, Chenal A, Gillet D, Pereira Y, Manich M (2004). Interaction between the two subdomains of the C-terminal part of the botulinum neurotoxin A is essential for the generation of protective antibodies.. Febs Letters.

[20] Volland H, Lamourette P, Nevers MC, Mazuet C, Ezan E (2008). A sensitive sandwich enzyme immunoassay for free or complexed Clostridium botulinum neurotoxin type A.. Journal of Immunological Methods.

[21] Lamoyi E, Nisonoff A (1983). Preparation of F(Ab')2 Fragments from Mouse Igg of Various Subclasses.. Journal of Immunological Methods.

[22] Hatheway CL, Dang C, Jankovic J, Hallett M (1994). Immunogenicity of neurotoxins of *Clostridium Botulinum* in therapy with botulinum toxin.. MarcelDekker, N.Y.

[23] Hill KK, Smith TJ, Helma CH, Ticknor LO, Foley BT (2007). Genetic diversity among botulinum neurotoxin-producing clostridial strains.. Journal of Bacteriology.

[24] Carter AT, Paul CJ, Mason DR, Twine SM, Alston MJ (2009). Independent evolution of neurotoxin and flagellar genetic loci in proteolytic Clostridium botulinum.. Bmc Genomics.

[25] Dover N, Barash JR, Arnon SS (2009). Novel Clostridium botulinum Toxin Gene Arrangement with Subtype A5 and Partial Subtype B3 Botulinum Neurotoxin Genes.. Journal of Clinical Microbiology.

[26] Smith TJ, Lou J, Geren IN, Forsyth CM, Tsai R (2005). Sequence variation within botulinum neurotoxin serotypes impacts antibody binding and neutralizaiion.. Infection and Immunity.

[27] Smith LA (1998). Development of recombinant vaccines for botulinum neurotoxin.. Toxicon.

[28] Clayton MA, Clayton JM, Brown DR, Middlebrook JL (1995). Protective Vaccination with A Recombinant Fragment of Clostridium-Botulinum Neurotoxin Serotype A Expressed from A Synthetic Gene in Escherichia-Coli.. Infection and Immunity.

[29] Sheridan RE, Deshpande SS, Amersdorfer P, Marks JD, Smith T (2001). Anomalous enhancement of botulinum toxin type A neurotoxicity in the presence of antitoxin.. Toxicon.

[30] Arnon SS, DasGupta BR (1993). Clinical trial of human botulism immune globulin.. Botulinum and Tetanus Neurotoxins: Neurotransmission and Biomedical Aspects.Plenum Press, N.Y.

[31] Arndt JW, Jacobson MJ, Abola EE, Forsyth CM, Tepp WH (2006). A structural perspective of the sequence variability within botulinum neurotoxin subtypes A1-A4.. Journal of Molecular Biology.

[32] Knauf MJ, Bell DP, Hirtzer P, Luo ZP, Young JD (1988). Relationship of Effective Molecular-Size to Systemic Clearance in Rats of Recombinant Interleukin-2 Chemically Modified with Water-Soluble Polymers.. Journal of Biological Chemistry.

[33] Lobo ED, Hansen RJ, Balthasar JP (2004). Antibody pharmacokinetics and pharmacodynamics.. Journal of Pharmaceutical Sciences.

[34] Batra SK, Jain M, Wittel UA, Chauhan SC, Colcher D (2002). Pharmacokinetics and biodistribution of genetically engineered antibodies.. Current Opinion in Biotechnology.

