# Supplementary figures and images for: Characterization of Botulinum Neurotoxin Type A Neutralizing Monoclonal Antibodies and Influence of Their Half-Lives on Therapeutic Activity
Source: PLoS One. 2010 Aug 26;5(8):e12416. doi: 10.1371/journal.pone.0012416 (PMC2928723; doi:10.1371/journal.pone.0012416)

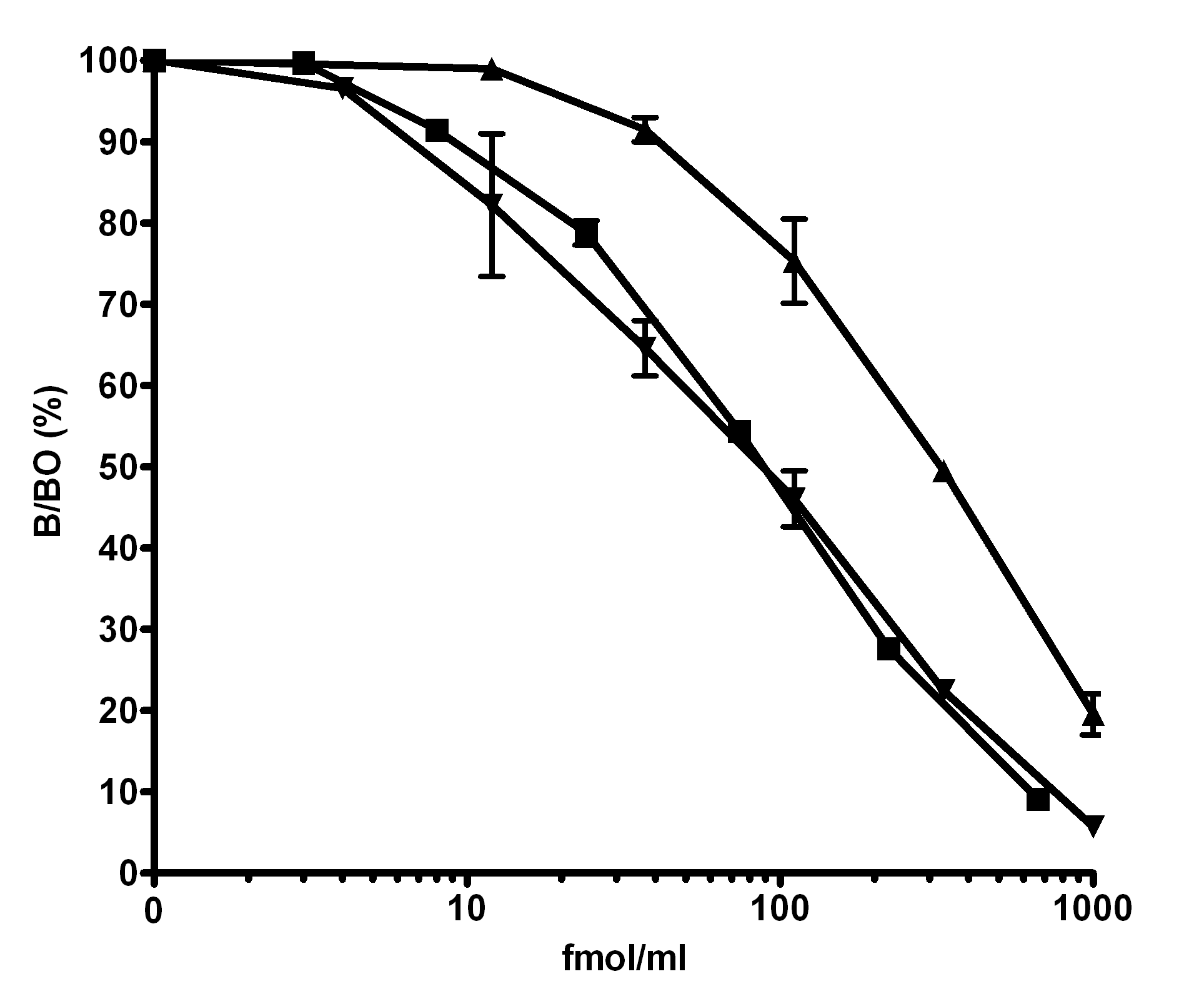

Supplement: Figure S1 — Examples of standard curves obtained for competitive sandwich assay of TA12 species (mAb, F(ab')2 or PEG-F(ab')2) in diluted plasma. ▪: TA12 mAb; ▾: TA12 F(ab')2; ▴: TA12 PEG-F(ab')2. The competitive sandwich assay is performed in the presence of a mAb tracer and another unlabeled mAb or fragment, both directed against the same epitope. There was competitive binding to the recombinant Hc BoNT/A1 immobilized on the plate by the capture mAb. This reaction leads to a signal decrease proportional to the competitor concentration. To perform this assay, 50 µl of recombinant Hc BoNT/A1 (2 ng/ml), 50 µl of TA12-acetylcholinesterase (AChE) and 100 µl of different concentrations of TA12 species (mAb, F(ab')2 or PEG-F(ab')2) were added to plates coated with TA5. The TA12 species were dilutions were made in diluted mouse plasma (1/20). After 18-h incubation at 4°C and washing of the plate, solid phase-bound AChE activity was revealed by the addition of 200 µl of Ellman's reagent for a 1-h reaction. The absorbances of the wells were then measured at 414 nm. (0.81 MB TIF) [file pone.0012416.s001.tif]
