# Supplementary material for: Characterization of Botulinum Neurotoxin Type A Neutralizing Monoclonal Antibodies and Influence of Their Half-Lives on Therapeutic Activity
Source: PLoS One. 2010 Aug 26;5(8):e12416. doi: 10.1371/journal.pone.0012416 (PMC2928723; doi:10.1371/journal.pone.0012416)
Supplement: Table S1 — Signals obtained for monoclonal antibody complementary tests. +++: Signals >1 absorbance unit; ++: Signals between 1 and 0.5 absorbance unit; +: Signals between 0.5 and 0.1 absorbance unit; +/−: Signals <0.1 absorbance unit; White squares: No specific signal. The simultaneous binding of the different mAbs to BoNT/A was analyzed in immunometric tests, with one mAb immobilized on the solid phase while the other was used tracer. Experiments were performed as follows: 10 ng/ml BoNT/A and 100 µl of a 100 ng/ml mAb tracer solution were added to microtitre plate wells coated with one of the mAbs. After 18-h incubation at 4°C, the plates were washed and the mAb tracer signal was measured (colorimetric signal). (0.07 MB DOC) [file pone.0012416.s002.doc]

**Table S1. Signals obtained for monoclonal antibody complementary tests**

| **Capture mAbs** | **Biotin-labelled mAbs** | | | | | | | | | | | | | |
| --- | --- | --- | --- | --- | --- | --- | --- | --- | --- | --- | --- | --- | --- | --- |
|  | **TA1** | **TA2** | **TA4** | **TA5** | **TA7** | **TA9** | **TA10** | **TA11** | **TA12** | **TA13** | **TA14** | **TA15** | **TA16** | **TA17** |
| TA1 |  | + | +++ |  | +++ | + | +/- | + | ++ | ++ | ++ |  | ++ | ++ |
| TA2 | + |  | ++ | + | ++ |  |  |  |  | ++ |  | + | + | ++ |
| TA4 | + | + |  | + |  | + |  | + | + |  | + | + | + |  |
| TA5 |  | + | +++ |  | +++ | + | +/- | + | ++ | +++ | ++ |  | ++ | ++ |
| TA7 | + | +/- |  | + |  | +/- |  | +/- | + |  | + | + | + |  |
| TA9 | ++ |  | ++ | ++ | ++ |  | + |  | + | ++ | ++ | + | ++ | ++ |
| TA10 | + |  | ++ | + | ++ | + |  |  |  | ++ | +/- | + | + | + |
| TA11 | + |  | ++ | + | ++ |  |  |  |  | ++ |  | + | + | ++ |
| TA12 | ++ |  | ++ | + | ++ | + |  |  |  | ++ |  | + | +/- | ++ |
| TA13 | + | + |  | + |  | +/- |  | + | + |  | + | + | + |  |
| TA14 | ++ |  | ++ | + | ++ | + |  |  |  | ++ |  | + | + | ++ |
| TA15 |  | + | ++ |  | ++ | + | +/- | + | + | ++ | +++ |  | ++ | ++ |
| TA16 | ++ | + | ++ | + | ++ | + | +/- | + | +/- | ++ | + | + |  | ++ |
| TA17 | + | + |  | + |  | +/- |  | + | + |  | + | + | + |  |

+++ : Signals >1 absorbance unit; ++ : Signals between 1 and 0.5 absorbance unit; + : Signals between 0.5 and 0.1 absorbance unit; +/- : Signals < 0.1 absorbance unit; White squares: No specific signal.

The simultaneous binding of the different mAbs to BoNT/A was analyzed in immunometric tests, with one mAb immobilized on the solid phase while the other was used tracer. Experiments were performed as follows: 10 ng/ml BoNT/A and 100 µl of a 100 ng/ml mAb tracer solution were added to microtitre plate wells coated with one of the mAbs. After 18-h incubation at 4°C, the plates were washed and the mAb tracer signal was measured (colorimetric signal).
