# Supplementary material for: Characterization of Botulinum Neurotoxin Type A Neutralizing Monoclonal Antibodies and Influence of Their Half-Lives on Therapeutic Activity
Source: PLoS One. 2010 Aug 26;5(8):e12416. doi: 10.1371/journal.pone.0012416 (PMC2928723; doi:10.1371/journal.pone.0012416)
Supplement: Table S2 — Toxin calibration. Toxin preparations were diluted in phosphate/gelatin buffer to obtain 5 estimated MLD/mouse and were checked as follows: 500 µl of toxin dilution were injected intraperitoneally into each mouse of a group of 3 to 4 mice. Results are expressed as the number of dead mice versus the total number of mice and are from 8 to 15 independent experiments. (0.03 MB DOC) [file pone.0012416.s003.doc]

**Table S2.** Toxin calibration

| Dilution | Pure | 1/5 | 1/10 |
| --- | --- | --- | --- |
| MLD/mouse | 5 | 1 | 0.5 |
| BoNT/A1 | 13/15 | 7/15 | 0/13 |
| BoNT/A2 | 9/10 | 6/10 | 0/10 |
| BoNT/A3 | 7/8 | 5/8 | 0/8 |

Toxin preparations were diluted in phosphate/gelatin buffer to obtain 5 estimated MLD/mouse and were checked as follows: 500 µl of toxin dilution were injected intraperitoneally into each mouse of a group of 3 to 4 mice. Results are expressed as the number of dead mice versus the total number of mice and are from 8 to 15 independent experiments.
